# Deep Learning–Based Prediction of Freezing of Gait in Parkinson's Disease With the Ensemble Channel Selection Approach

**DOI:** 10.1002/brb3.70206

**Published:** 2024-12-31

**Authors:** Sara Abbasi, Khosro Rezaee

**Affiliations:** ^1^ Department of Biomedical Engineering Islamic Azad University of Mashhad Mashhad Iran; ^2^ Department of Biomedical Engineering Meybod University Meybod Iran

**Keywords:** bidirectional long short‐term memory, bottleneck attention module, channel selection, ensembling, freezing of gait, Parkinson's disease

## Abstract

**Purpose:**

A debilitating and poorly understood symptom of Parkinson's disease (PD) is freezing of gait (FoG), which increases the risk of falling. Clinical evaluations of FoG, relying on patients’ subjective reports and manual examinations by specialists, are unreliable, and most detection methods are influenced by subject‐specific factors.

**Method:**

To address this, we developed a novel algorithm for detecting FoG events based on movement signals. To enhance efficiency, we propose a novel architecture integrating a bottleneck attention module into a standard bidirectional long short‐term memory network (BiLSTM). This architecture, adaptable to a convolution bottleneck attention–BiLSTM (CBA‐BiLSTM), classifies signals using data from ankle, leg, and trunk sensors.

**Finding:**

Given three movement directions from three locations, we reduce computational complexity in two phases: selecting optimal channels through ensemble learning followed by feature reduction using attention mapping. In FoG event detection tests, performance improved significantly compared to control groups and existing methods, achieving 99.88% accuracy with only two channels.

**Conclusion:**

The reduced computational complexity enables real‐time monitoring. Our approach demonstrates substantial improvements in classification results compared to traditional deep learning methods.

## Graphical abstract

The figure depicts a pipeline for detecting freezing of gait (FoG) in Parkinson's Disease (PD). Sensor data is collected, pre‐processed (normalization and shuffling), and filtered through channel selection. A CBA‐BiLSTM model classifies FoG events and differentiates PD from non‐PD cases, enabling accurate detection and intervention.

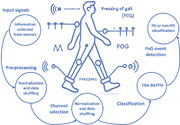

## 1 Introduction

Parkinson's disease (PD) is an irreversible neurodegenerative disturbance characterized by postural abnormalities, bradykinesia, rigidity, resting tremor, and freezing of gait (FoG) (Kalia and Lang 2016). The number of people suffering from PD in 2016 was estimated at 6.1 million (Dorsey et al. 2018). The number of PD cases per 100,000 people varies between 35.8 and 12.500, according to various estimates (Khatri et al. 2020; Shi et al. 2022). Age is significantly associated with increased PD incidence (Trist et al. 2019). The prevalence of PD in older adults is estimated to be between 1.3% and 3.0% (Riedel et al. 2016). Between 2005 and 2015, PD diagnoses increased by 31.6%, and its prevalence increased by 21.0%. These shifting meanings have propelled PD to the forefront of the neurodegenerative disease epidemic, making it the most difficult health concern facing aging populations.

PD causes severe motor symptoms, which result in significant impairments in everyday activities such as walking and standing still (Maas and van de Warrenburg 2021). In 50% of PD patients, FoG is the most severe movement disorder that impairs their quality of life (Barbe et al. 2020; Shah et al. 2018). FoG is characterized by the lack of forward leg movement, which may be absent or greatly diminished during certain periods, but it is always transient (Kim et al. 2024; Kung et al. 2023).

According to a recent study, FoG can also occur in people with intermediate and late‐stage PD (Okuma 2014). FoG is typically observed in patients with severe PD (Milane et al. 2023). The severe impairment of mobility and independence caused by PD often results in catastrophic injuries caused by falls. There are a variety of causes of FoG, including internal and external circumstances, such as turning, space, walking in a confined area, doing multiple tasks, and stressful events (such as being immobilized upon arrival at the destination). FoG episodes typically last a few seconds, but sometimes they last several minutes (Huang et al. 2018). Freezing consequences have been treated differently from the therapy intended to prevent them. PD patients can benefit from cueing in addition to medication when dealing with frontotemporal dementia or avoiding falls (Snijders et al. 2008; Nonnekes et al. 2015; Kim et al. 2022).

In recent years, this design has been practical because of the decrease in the price of single‐board central processing units for wearable electronics. A large number of works currently use inertial measurement units (IMUs) to generate data because they generate accurate data, and they can be worn by patients without interfering with their normal activities in the form of accelerometers or gyroscopes. Besides, FoG and gait issues have been diagnosed using vision‐oriented techniques (Morris, Martin, and Schenkman 2010; Sun et al. 2018). Vision‐based approaches, however, have shown less satisfactory results than IMU‐based approaches. Furthermore, these approaches are limited by privacy and security concerns.

To recognize FoG events, conventional machine learning (ML) techniques require extensive domain knowledge, preprocessing, and feature engineering. No one sign or combination of symptoms can fully describe a freezing episode due to its complexity and diversity. Deep learning (DL) methods have been employed to detect FoG without artificial attributes in recent research (Shi et al. 2022; Hu et al. 2019; Sigcha et al. 2020; Bikias et al. 2021; Shalin et al. 2021; Hu et al. 2021; Habib et al. 2024). Sensor data were analyzed using DL models instead of ML models. The convolutional neural networks (CNNs) algorithm captures complex and diversified features without requiring a lot of data knowledge, unlike conventional ML algorithms. As a result, it has become a sought‐after tool for analyzing clinical data.

There have been a variety of alternative feature extraction and data reduction techniques proposed in recent years for a broad range of applications. Many of these applications are based on ML and DL algorithms. There have been several studies that help to understand the discrimination of received signals in gait movements (Skaramagkas et al. 2023; Beigi et al. 2023; Önder et al. 2023). However, many researchers have faced major challenges in uncovering FoGs through ML techniques. During this process, handcrafted and deep structures are used to extract features through traditional and innovative methods. To enhance general data analysis operations (efficiency), feature extraction and data reduction techniques are used to increase feature description (efficiency) or minimize computational complexity. As there are several strategies to analyze signals or data, we examine bottleneck attention within the newly developed and incredibly promising DL framework (Nagrani et al. 2021).

This article proposes an automated method for evaluating non‐stationary 2D acceleration data that reduces false positive and false negative PD diagnoses. We investigated different acceleration signal types instead of using comparable methods to evaluate PD. To detect PD using acceleration signals, a hybrid model was developed to predict PD onset. A two‐class PD classification system can be built using a new model to interpret acceleration data. As far as we know, this is the first application of a bottleneck attention module–inspired architecture based on a plain bidirectional long short‐term memory network (BiLSTM) to PD classification. Accelerometers can compare the acceleration data of several PD signals to find patterns. In order to detect PD, the novel classifier uses the bottleneck attention module.

Moreover, for assessing channel weights, we propose a *k*‐nearest neighbors (*k*‐NN) classifier based on supervised learning and an innovative channel selection method. With the enhanced channel selector and bottleneck attention module–inspired architecture based on plain bidirectional LSTM (BiLSTM) or the convolution bottleneck attention–BiLSTM (CBA‐BiLSTM), statistical results of significant characteristics are integrated using an objective ranking approach. This approach rather than relying on incompetent and biased expert knowledge, focuses on leveraging objective and data‐driven methodologies to ensure accuracy and reliability. Accordingly, this work aims to develop, using acquired sensor data, a scalable and accurate approach to investigating PD through accelerated signals.

Our bottleneck attention module–based model for FoG detection presents two key innovations. First, it introduces a novel architecture that integrates a bottleneck attention module into a standard BiLSTM. This integration allows the model to focus on the most relevant features within the movement signal data, leading to more accurate FoG prediction. Second, this model represents the first documented application of a CBA‐BiLSTM specifically for PD classification and FoG detection. This novel approach has the potential to significantly improve the accuracy of FoG prediction compared to traditional methods. Indeed, this method distinguishes itself from other quantitative approaches in the field, offering significant improvements in both accuracy and practicality. It is markedly superior, providing a more reliable and efficient solution for detecting FoG in PD patients. Our work contributes to:

1. It is the first‐time signals brought about by patients’ motion are identified by a combined effective model. We therefore offer a bottleneck attention module–inspired architecture based on plain BiLSTM that categorizes signals based on data collected from ankle, leg, and trunk sensors.
2. Due to the three movement directions that are received from three locations, we reduce the computational complexity in two stages: One is to select the highest efficient channels using ensemble learning, and the second is to reduce the features using attention mapping.
3. In the automated model based on movement signals for FoG and PD detection, channel selection and classifier choice are crucial. Its main advantage is that it does not require signal windowing. Additionally, the improved model based on training data can provide individual monitoring and FoG detection. The model is also able to predict PD progression and can serve as a tool to detect early signs of PD. Furthermore, it is a cost‐effective and non‐invasive method for detecting FoG and PD.

As for the rest of the article, it is arranged as follows: Section 2 provides related work. In Section 3, we present a model for identifying PD based on acceleration signals. Section 4 presents datasets, outcomes, and discussion. Toward the end of the article, the study's conclusions and potential future directions are discussed.

## 2 Related Work

This section describes cutting‐edge research conducted to identify and forecast FoG. Shi et al. (2022) applied the continuous wavelet transform (CWT) to signals from IMUs connected to the lower limbs of 63 PD patients with FoG to evaluate the time‐frequency domain utilization. CNNs and Bayesian optimization were used to define FoG identification hyperparameters. According to the results, the subject‐independent model received a geometric mean of 90.7% and an F1 score of 91.1%.

To interpret data from a single accelerometer worn on a participant's wrist, Sigcha et al. (2020) recommended using a CNN combined with an LSTM (CNN‐LSTM) deep neural network model. Twenty‐one individuals participated in the study, and information was collected from them at home to optimize FoG episode frequency. According to leave‐one‐subject‐out (LOSO) validation, the CNN‐LSTM model provides the most effective results when three past spectral windows are stacked on the current window, with mean sensitivity, specificity, and geometric mean values of 87.1%.

For the purpose of detecting FoG with a DL approach, Bikias et al. (2021) collected linear and angular acceleration data from wrist. Through LOSO and 10‐fold cross‐validation (CV), data collected during the data collection procedure was used to train a CNN model. According to the results, the LOSO‐CV had a sensitivity of 83% and a specificity of 86%. Ten‐fold CV results indicate 90% and 88% specificity and sensitivity.

Using recurrent neural networks (RNNs) and LSTMs, Shalin et al. (2021) developed a DL model to predict FoG in PD patients. One‐second forecasts were successful 94.7% of the time, 3‐s forecasts were successful 82.9% of the time, and 5‐s forecasts were reliable 68.1% of the time.

Deep recurrent neural networks (DRNNs) were used to build recognition models that detected long‐range correlations between sequences of varying durations in Murad and Pyun (2017). LSTM‐DRNNs serve as the foundation for the proposed approaches, which include cascaded, single‐directional, and bidirectional approaches. In the UCI dataset, unidirectional deep RNN improved accuracy to 96.7%, 96.8%, 96.7%, and 0.96%. For the USC‐HAD dataset, unidirectional deep RNN improved accuracy to 97.2%, 97.3%, 97.5%, and 0.97%. On the OPPORTUNITY dataset, bidirectional deep RNN had error rates of 92.7%, 86.7%, 83.5%, and 0.92%, respectively. On the Daphnet dataset, cascaded deep RNNs achieved 94.1% accuracy, 84.7% accuracy, 78.5% accuracy, and 0.93% accuracy. On the Skoda dataset, cascaded deep RNN networks performed 92.6%, 93.1%, 92.6%, and 0.92, respectively. All metrics were evaluated, including accuracy, average accuracy, average recall, and F1 score.

FoG detection was developed by Xia et al. (2018) based on CNN trained on a 2D acceleration signal. According to the independent subject, accuracy, precision, and specificity averaged 80.7%, 69.29%, and 90.0%, respectively. To gather data, 10 volunteers were fitted with accelerometers at 3 distinct locations on their bodies. The raw accelerometer data were cleaned and segmented with a window size of 4 s before being used as input to the structure.

According to San‐Segundo et al. (2019), the authors deploy a DNN with a 4‐s frame and four different feature sets to identify FoG. It has been suggested to use the multilayer perceptron (MLP)‐layered CNN model. A max‐pooling layer, two convolutional layers, and three fully connected layers are used for feature extraction and categorization, respectively. A sensitivity of 93.1% and a specificity of 75% were obtained.

Additionally, Camps et al. (2017) used DL techniques and image analysis to identify FoG events. 1D‐ConvNet‐based technique was developed for this application. In their study, the specificity was 78% and the sensitivity was 88.6%.

In order to detect FoG, Camps et al. (2018) developed a 1D 8‐layer CNN. Nine‐channel tri‐axial IMUs (magnetometer, gyroscope, and accelerometer) were worn on the left wrist area of 21 individuals with PD to train the model during walking tests at home. Fast Fourier transform (FFT) was used to convert each 2.56‐s data frame into the frequency domain. For a typical sample, the magnitude of each FFT window was averaged with the magnitude of the previous window. After further processing, the authors augmented the data to resolve the discrepancy. The model outperformed its shallow ML predecessors by a wide margin. It achieved a sensitivity of 91.9%, a specificity of 89.5%, and a geometric mean accuracy of 90.6%.

An approach described in Kim et al. (2018) used CNNs with two types of data: temporal and frequency domain information with a 2.5‐s frame to detect FoG episodes. A gyroscope and accelerometer sensor on a smart‐phone in the patient's pocket was used to extract the aforementioned features. F1 score, sensitivity, and specificity were all 90.1%.

Samà et al. (2018) proposed novel features to detect FoG in real‐world contexts using accelerometers. They evaluated a variety of window widths and ML algorithms to train the various feature sets. As a result of using the suggested technique, they identified FoG at patients’ residences with a sensitivity of 91.7% and a specificity of 87.4%, thereby improving the outcomes of previous approaches by 5%–11% and presenting a more balanced false positive and false negative rate.

Naghavi and Wade (2019) conducted a novel approach to identify the optimal placement of sensors, optimal orientation of sensors, optimum length of sampling windows, and ideal FoG prediction parameters. In a variety of laboratory settings, patients with PD experiencing FoG helped characterize the device's performance. Using 120 different setups based on their findings, a shank sensor and a sample entropy derived from the horizontal forward axis with a window length of 2 s were used to predict FoG with an average predictivity of 88.8%, a sensitivity of 92.5%, and a specificity of 89.0%.

To reduce the impact of imbalanced datasets on model training for PD detection, Li et al. (Singh and Tripathi 2023) presented squeeze‐and‐excitation blocks and attention strategies. In a CV evaluation, the sensitivity and specificity were increased by 0.017 and 0.045, respectively, compared to the previous most accurate results. A LOSO CV evaluation resulted in a 1.9% decrease in the equal error rate. It can be concluded from these results that the proposed architecture possesses excellent detection performance and appropriate operating efficiency. It detects 256 segments in less than 0.52 ms.

Habib et al. (2024) developed a DL framework for predicting PD using Wi‐Fi‐based Channel State Information (CSI) to detect FoG. They introduced two models, Deep Dual Attention Neural Network and Bi‐LSTM Neural Network, which were trained on data from a wireless sensor network. These models used dual self‐attention for feature extraction and a weighted fusion approach, followed by tree growth optimization for feature selection. Achieving 98.66% accuracy, this method outperformed existing techniques. Despite its strengths, such as high accuracy and an innovative approach, the study faced limitations like a small sample size and complexity.

Singh and Tripathi (2023) employed ML models to diagnose PD using gait sensor signals. They compared two experiments, one without feature selection and the other using the recursive feature elimination algorithm, achieving a high accuracy of 96.1% with the ensemble voting classifier. The study's strengths included high accuracy and optimized feature selection, whereas its weaknesses involved potential limitations in generalizability and computational complexity. The dataset details were not fully disclosed, limiting insights into its diversity and representativeness.

Bajpai, Khare, and Joshi (2023) presented an ensemble model combining EEG and IMU data through two neural networks, EEGFoGNet and IMUFoGNet, achieving a top accuracy of 92.1% at a 1‐s prediction horizon. Strengths included the integration of multimodal data and the use of transfer learning for personalization, enhancing prediction accuracy. However, the complexity of the model and the requirement for comprehensive monitoring systems were noted weaknesses. The dataset used involved EEG and IMU data, but specific details about its size and diversity were lacking, affecting the assessment of its applicability.

Ouyang et al. (2023) proposed an autoregressive predictive model followed by a support vector machine (SVM) classifier to predict FoG using accelerometer and gyroscope data, achieving an accuracy of 85.08%. Strengths included robust performance and consistency across subjects, with an effective feature selection process. Weaknesses involved a limited prediction horizon and reliance on specific sensor types. The dataset consisted of time‐series data from wearable sensors, but detailed information about its size and diversity was not provided, limiting the evaluation of its generalizability.

Despite significant advances in detecting FoG in PD using various ML and DL techniques, previous studies have often been limited by small sample sizes, complex methodologies, and specific hardware requirements. For instance, methods leveraging IMUs, CNNs, and RNNs have achieved high accuracy but often require intricate setups and extensive data preprocessing. In contrast, the plain BiLSTM and the CBA‐BiLSTM offer superior performance by effectively capturing temporal dependencies and enhancing feature extraction through attention mechanisms. These models simplify the detection process and improve robustness, making them more practical for real‐world applications. Their ability to balance high accuracy with operational efficiency marks a significant improvement over prior approaches, addressing key limitations and advancing the field of FoG detection for PD.

## 3 Methodology

**FIGURE 1:**
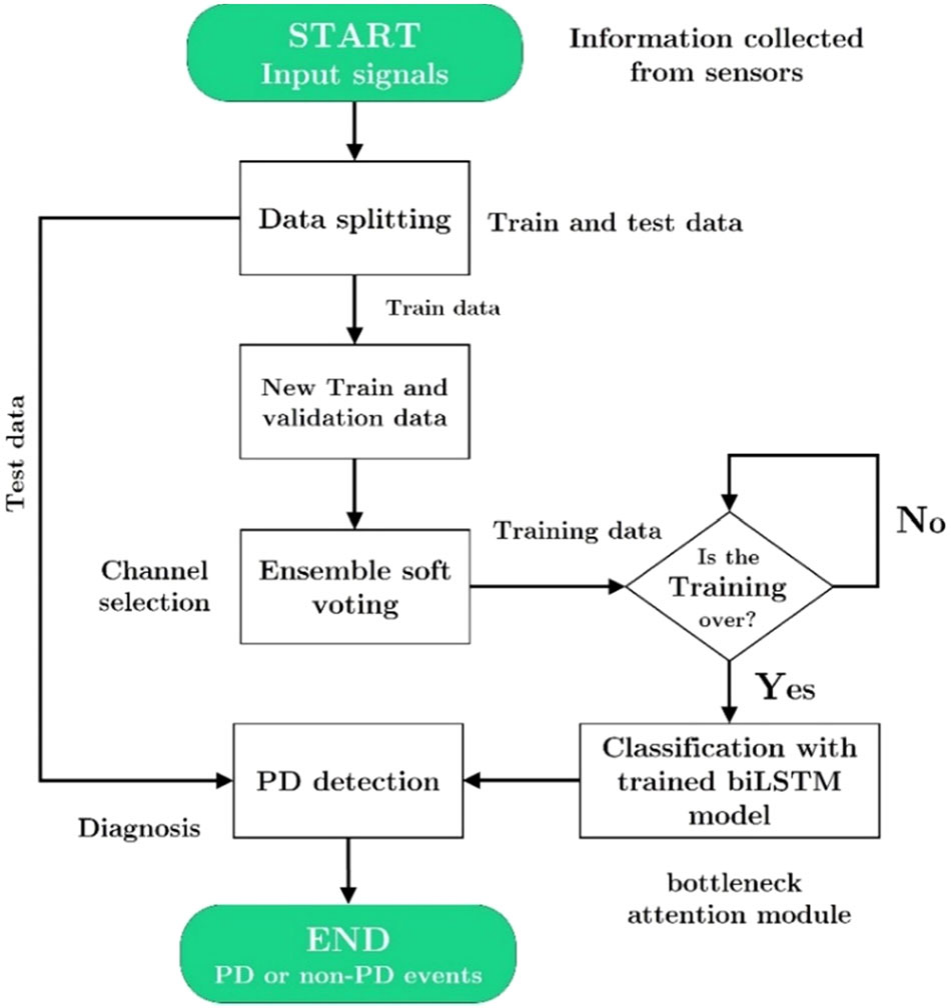
The diagram shows how FoG events are detected and then used to classify signals as PD. biLSTM, bidirectional long short‐term memory network; PD, Parkinson's disease.

Using the introduced method, Figure 1 illustrates how our model recognizes FoG events in input signals. In the following sections, we describe this method in more detail.

### 3.1 Signal Preprocessing

By taking different channels of movement, the signals have already been created (Li et al. 2020). We did not employ the windowing technique to analyze walking signals; as a result, we received and processed walking data from three sensors. In order to avoid overtraining the automatic classification model, we normalized the new training data. Although the samples themselves are randomly mixed, the positioning of the labels may differ based on the classes represented by the samples. A learning system can be trained to recognize a certain type of channel by merging samples during validation. It can also modify the labels corresponding to sample locations. This approach enhances classification precision by reducing overfitting likelihood (Bächlin et al. 2010). During preprocessing, training data are normalized because each channel employs its own set of characteristics and is naturally highly dispersed. It is also possible to reduce computational costs by standardizing channel analysis in addition to saving time and decreasing computational complexity. Normalizing the range (0, 1) is appropriate for min–max normalization.

### 3.2 Channel Selection

In order to select channels of gait walking, receiver operating characteristics (ROC), Wilcoxon procedure, and signal‐to‐noise ratio (SNR) are utilized. Ascertaining the accuracy and inaccuracy of test results using ROC curves is the first method. On the basis of ROC, we can calculate the area under the curve (AUC) using the following equation (Liu et al. 2022):

(1) $$\mathrm{if}\;t\in \left(0,1\right)\to AUC=\int_{0}^{1}T_{1}\left(\frac{1}{T_{2}}\left(t\right)\right)dt$$

In this respect, *T* is an extension of *F_i_*, in which *F*_1_(*x*) and *F*_2_(*x*) are two independent distribution functions (*x*). The channel selection vector only includes channels with a high AUC.

The Mann–Whitney test determines whether two populations are statistically equal in the same way as the Wilcoxon test. If we accept the null hypothesis, the distribution functions of the two populations are equal; if we accept the alternative, they are different. This test is popular because it does not require an a priori hypothesis about the difference among the samples. Wilcoxon signed‐rank test is conducted based on several steps. In the first step, we will join both sets of data and rank them based on the total number of observations. By adding the rankings of subgroups, Wilcoxon's statistic is calculated. In the third step, *p* values are generated using the whole distribution table, and these values are then used to draw conclusions. When selecting channels, the Wilcoxon test's absolute values of the standardized Wilcoxon statistic are superior to the test's Wilcoxon coefficients. Moreover, the SNR approach (Rezaee and Zolfaghari 2022) also compares the connectivity of channel labels:

(2) $$SNR_{FS}=\frac{\left(\mu_{1}-\mu_{2}\right)}{\left(\sigma_{1}+\sigma_{2}\right)}$$

where *μ*_1_ and *μ*_2_ indicate the sample mean, whereas *σ*_1_ and *σ*_2_ indicate the standard deviation. The former transports the channel labels, and the latter represents the *i*‐th characteristic vector. A model can be used to resolve the difficulty of selecting channels and categorizing gait events from human walking. During the training step, the attributes with the most votes are selected as the most significant.

**FIGURE 2:**
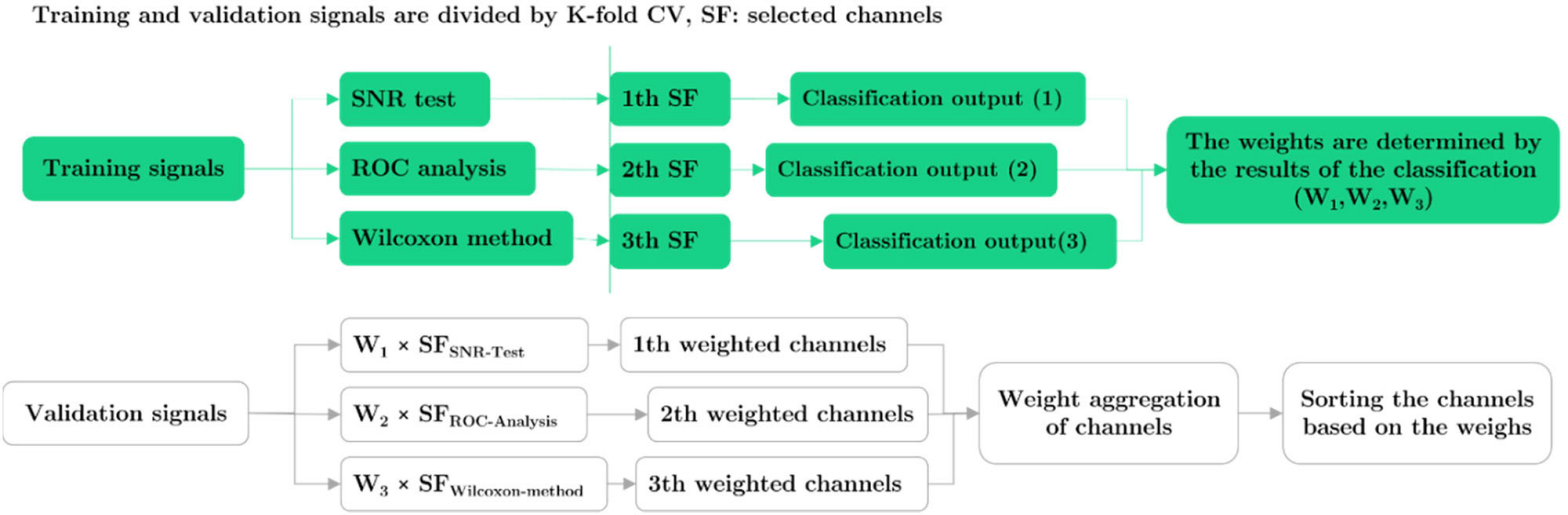
In our method of ensembling, three metrics are used to find the most advantageous channels, with the channel weight increasing the classification accuracy of the training data. CV, cross‐validation; ROC, receiver operating characteristic; SNR, signal‐to‐noise ratio.

In the process of selecting the best channels, the *k*‐NN algorithm acts as an effective classifier. The use of *k*‐NN, due to its simplicity and high accuracy, is an efficient and reliable method for selecting important channels, especially in large and complex datasets. Channels that have the greatest impact on classification accuracy are then selected. As a result of the K‐fold method, that is, CV = 10, the training part is divided into new, validation, and training sets, and the most frequent channel is used to represent the selected channel. This technique predicts the classification class using the principal channel, and *k* is the closest selected neighbor; it is efficient and provides appropriate results for large datasets. By using a query and *k*–*d* algorithm, Rezaee, Badiei, and Meshgini (2020) minimize computing burden and improve classification accuracy. A subtle trend of channel selection is shown in Figure 2 when training accuracy is taken into account.

### 3.3 Bottleneck Attention and BiLSTM

LSTM has been demonstrated to be useful in several areas, including data forecasting and FoG incident identification (Nagrani et al. 2021; Ashour et al. 2020; Chen et al. 2023). Thus, the *t*‐th representation of time in LSTM can be expressed abstractly and inclusively as follows:

(3)

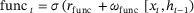

(4) $$i_{t}=\sigma \left(r_{i}+\omega_{i}\left[x_{r},h_{t-1}\right)\right.$$

(5) $$o_{t}=\sigma \left(r_{0}+\omega_{o}\left[x_{t},h_{t-1}\right)\right.$$

(6)

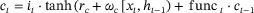

(7) $$h_{t}=o_{t}\cdot \tanh\left(c_{t}\right)$$

In this context, the inputs are represented as (*x_t_*), the hidden state is denoted as (*h_t_*), and the current state of the cell is called (*c_t_*). Other sigmoid‐shaped learning parameters include *ω_func_*, *ω_c_*, *ω_o_*, and *ω_i_*, along with *r_func_*, *r_c_*, *r_o_*, and *r_i_*. The output, denoted as Out*_t_*, of the biSTM model may be determined by integrating the forward *h^f^_t_* and backward *l^b^_t_* directions:

(8) $$h_{t}^{f}=\left(\to \right)\mathrm{LSTM}\left[x_{t},h_{t-1}\right]$$

(9) $$l_{t}^{b}=\left(\leftarrow \right)\mathrm{LSTM}\left[x_{t},h_{t+1}\right]$$

(10) $$Out_{t}=\left[h_{t}^{f};l_{t}^{b}\right]$$

Due to their inherent limitations in feature reduction and representation, particularly in terms of channel selection (see Section 3.2), these simplistic models require further modification to enhance their accuracy. The methodology employed in our study for automating FoG incident identification is rooted in the bottleneck attention module (Park et al. 2018). We have devised an effective architecture that incorporates a basic BiLSTM model. The design of this model incorporates two routes, namely, channel attention (*M_c_*) and spatial attention (*M_r_*), which are utilized to infer attention maps. These attention maps enhance feature reduction and representation inside the model.

#### 3.3.1 Attention Module

In this study, we utilize channel attention, a technique that involves three specific operations (global average pooling *F_gap_*, batch normalization, and fully connected *F_f_*) to exploit the inter‐channel interaction inside the channel branch. This is particularly relevant since each channel corresponds to a unique feature response. By using Equations (3)–(9) on the given input *x_t_* = [*x_t_* ^1^, *x_t_* ^2^, *x_t_* ^3^, …, $x_{t}^{\overset{\prime }{C}}$], we obtain the values of *h^f^_t_* = [*h_t_*_1_, *h_t_*_2_, *h_t_*_3_, …, *h_tc_*] and l*^b^_t_* = [*l_t_*_1_, *l_t_*_2_, *l_t_*_3_, …, *l_tC_*], along with *x_t_* ∈ $R^{\overset{\prime }{N}\times \overset{\prime }{M}\times \overset{\prime }{C}}$, *h_tC_* ∈ $R^{N\times M}$, and l*_tC_* ∈ $R^{N\times M}$. The *F_gap_* approach is employed for encoding global data in both the forward *h^f^_t_* and backward *l^b^_t_* directions:

(11) $$u_{c}\left(\to \right)=F_{gap}\left(h_{c}\right)={\left(N\times M\right)}^{-1}\sum_{j=1}^{N}\sum_{j=1}^{M}h_{C}\left(i,j\right)$$

(12) $$u_{c}\left(\leftarrow \right)=F_{g\mathrm{ap}}\left(l_{tC}\right)={\left(N\times M\right)}^{-1}\sum_{l=1}^{N}\sum_{j=1}^{M}l_{tC}\left(i,j\right)$$

Additionally, batch normalization and fully connected *F_f_* processes are applied to the resultant global features:

(13) $$S\left(\to \right)=Batch_{\mathrm{norm}}\left(F_{f}\left(U\right)\left(\to \right)\right)=Batch_{\mathrm{norm}}\left(\sigma \left[\omega_{2}\delta \left(U_{\omega_{2}}\left(\to \right)\right)\right]\right)$$

(14) $$S\left(\leftarrow \right)=Batch_{\mathrm{norm}}\left(F_{f}\left(U\right)\left(\leftarrow \right)\right)=Batch_{\mathrm{norm}}\;\left(\sigma \left[\mu_{2}\delta \left(U_{\mu_{1}}\left(\leftarrow \right)\right)\right]\right.$$

**FIGURE 3:**
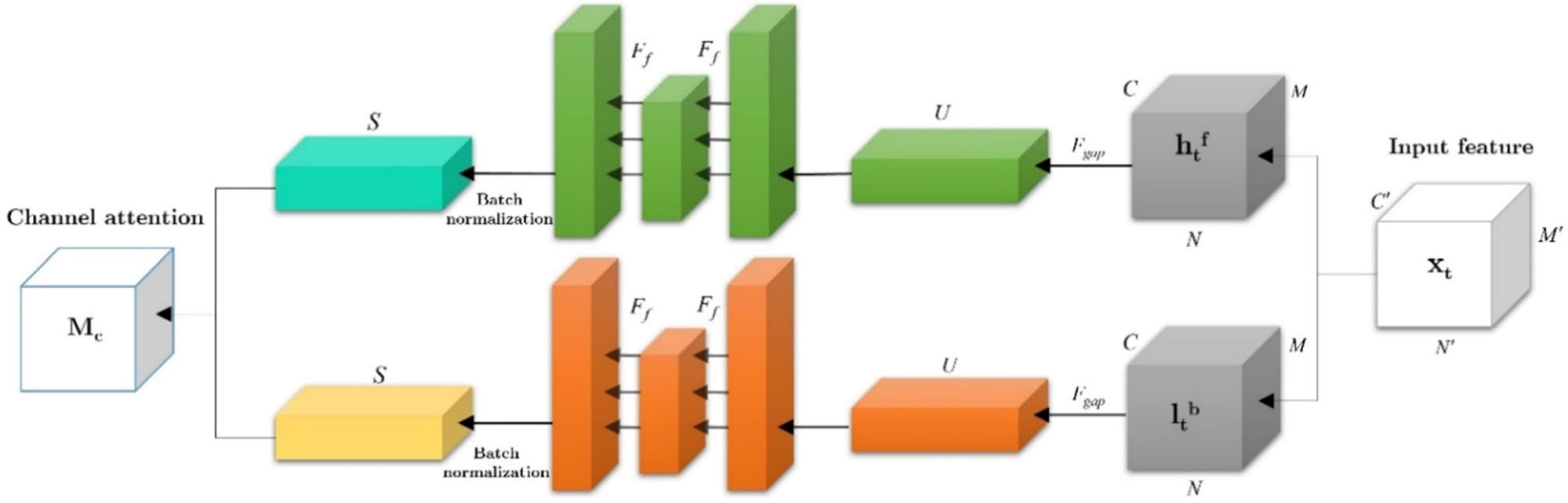
This figure outlines the concept of channel attention. The aforementioned structure encompasses three distinct processes, namely, global average pooling (*F_gap_*), fully connected (*F_f_*), and batch normalization.

*u_c_* (→) and *u_c_* (←) are the *C*‐th components of *U*(→), *U*(←). In addition, *S*(→), *S*(←) ∈ *R^C^* ^×1×1^ are the variables used in BiLSTM to denote the attention maps for both direction channels. As shown in Hu, Shen, and Sun (2018), the learning parameters (*δ*) for the rectified linear unit (ReLU) are *ω*_1_ ∈ *R* ^(^ *^C^* ^/^ *^r^* ^)×^ *^C^*, *ω*_2_ ∈ *R^C^* ^×(^ *^C^* ^/^ *^r^* ^)^, *μ*_1_ ∈ *R* ^(^ *^C^* ^/^ *^r^* ^)×^ *^C^*, and *μ*_2_ ∈ *R^C^* ^×(^ *^C^* ^/^ *^r^* ^)^. Figure 3 shows the results of using the provided data to develop channel attention *M_c_* (•) = [*S*(→); *S*(←)].Using spatial attention, it is possible to emphasize or de‐emphasize feature information in different regions in order to compensate for the inherent constraints of channel attention. In addition, this module incorporates three distinct processes, namely, 3 × 3 dilated convolution (*F_dc_*) (Yu and Koltun 2016), 1 × 1 convolution $F_{f^{1\times 1}}$, and batch normalization. Due to its enhanced effectiveness, dilated convolution with an expanded receptive field is preferred in the construction of spatial attributes. Channel capacity is reduced by 1 × 1 convolutions. The three operations that Out*_t_* experiences are as follows:

(15) $$V\left(\to \right)=Batch_{\mathrm{norm}}\;\left(F_{f^{1\times 1}}\left(F_{dc}\left(F_{dc}\left(F_{f^{1\times 1}}\left(h_{t}^{f}\right)\right)\right)\right)\right)$$

(16) $$V\left(\leftarrow \right)=Batch_{\mathrm{norm}}\;\left(F_{f^{1\times 1}}\left(F_{dc}\left(F_{dc}\left(F_{f^{1\times 1}}\left(l_{t}^{f}\right)\right)\right)\right)\right)$$

**FIGURE 4:**
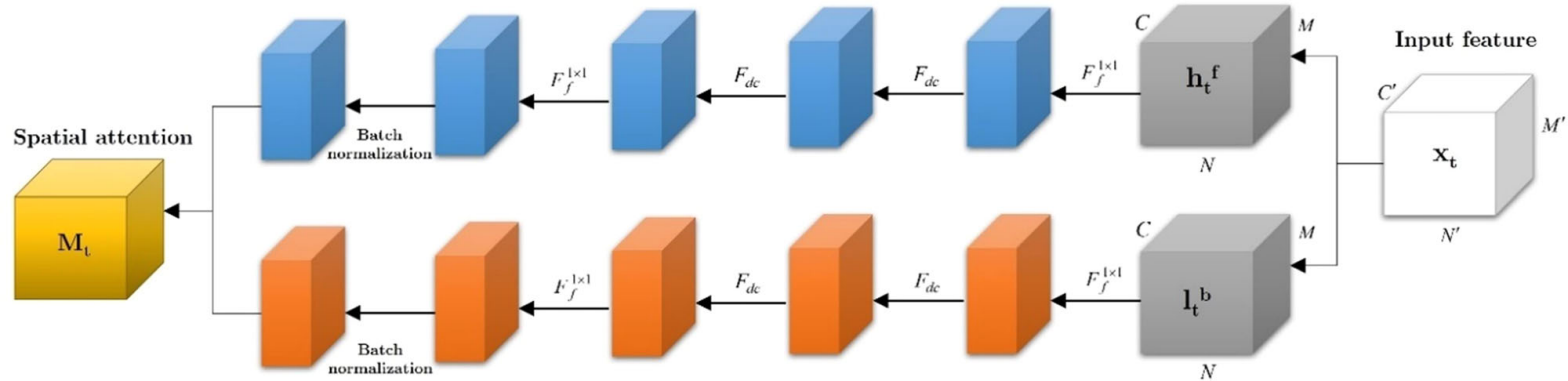
An illustration of spatial attention. It is mostly composed of three operations: batch normalization, 3 × 3 dilated convolution *F_dc_*, and 1 × 1 convolution $F_{f^{\;1\times 1}}$.

*S*(→) and *S*(←) ∈ *R* ^1×^ *^N^* ^×^ *^M^* are defined there. The spatial attention *M_s_* (•) = [*V*(→); *V*(←)] is also available in Figure 4.

#### 3.3.2 Channel and Spatial Attention Fusion

**FIGURE 5:**
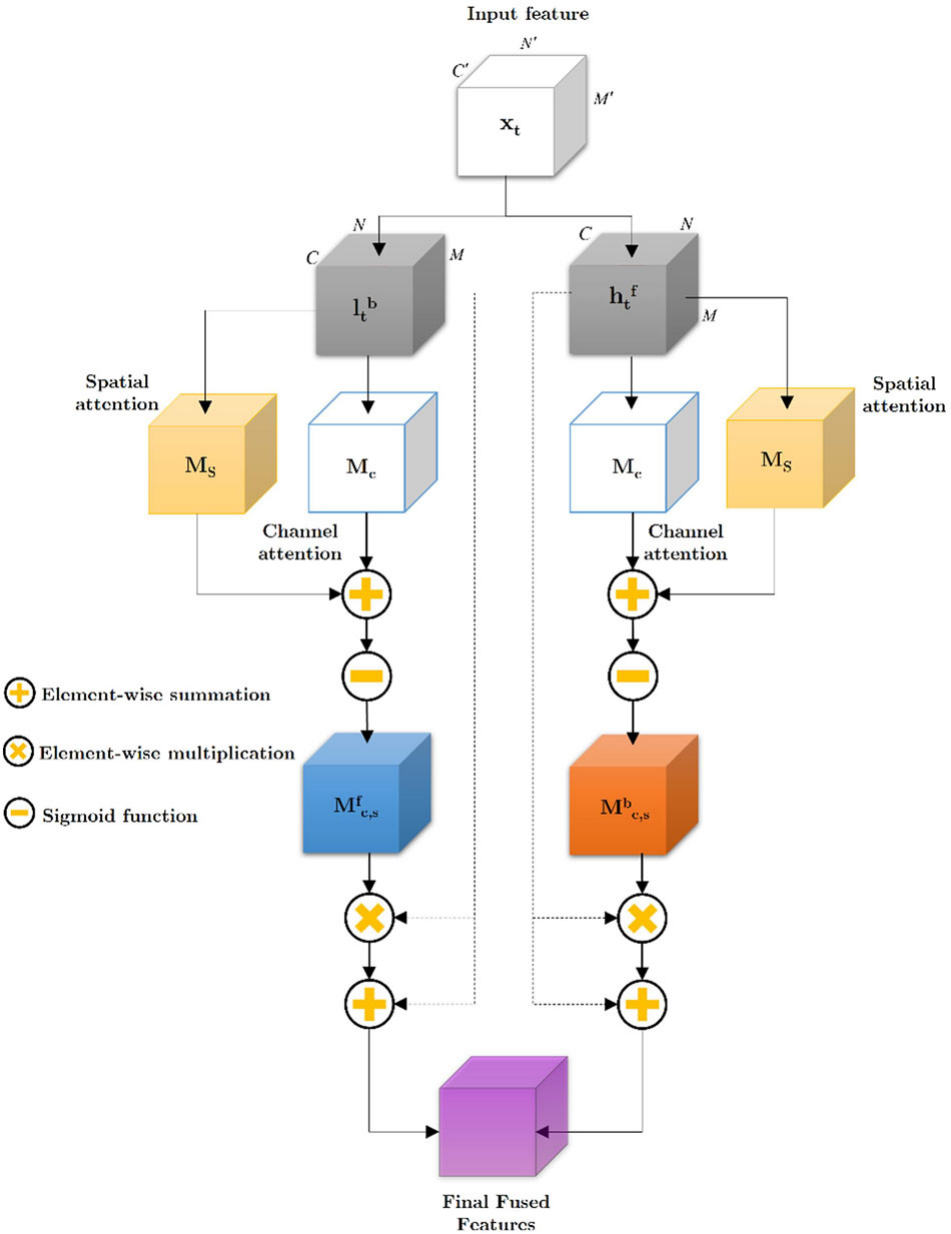
This figure depicts the bottleneck‐LSTM framework. The aforementioned architecture, constructed upon a conventional BiLSTM model, possesses the capability to deduce channel attention (*M_c_*) and spatial attention (*M_s_*) maps.

The fusion of channel attention *M_c_* ∈ *R^C^* ^×1×1^ and spatial attention *M_s_* ∈ *R* ^1×^ *^N^* ^×^ *^M^* is performed in the BiLSTM model. As shown in Figure 5, the model of bottleneck attention is based on BiLSTM.

This approach aims to create a bottleneck attention–BiLSTM module that is both efficient and powerful. Given that the two attention maps exhibit clear differences, we proceed to calculate a synthetic map, referred to as *M_c,s_*, by merging *M_c_* and *M_s_*, where they belong to the *R^C^* ^×^ *^N^* ^×^ *^M^*:

(17)

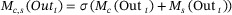




Next, the attention mechanism is employed alongside the residual learning approach to enhance gradient flow. The improved feature maps of the recommended BiLSTM bottleneck attention map is presented as follows:

(18)

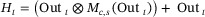




An element‐wise multiplication can be performed by the ⊗︀ operator.

#### 3.3.3 Convolutional Architecture

**FIGURE 6:**
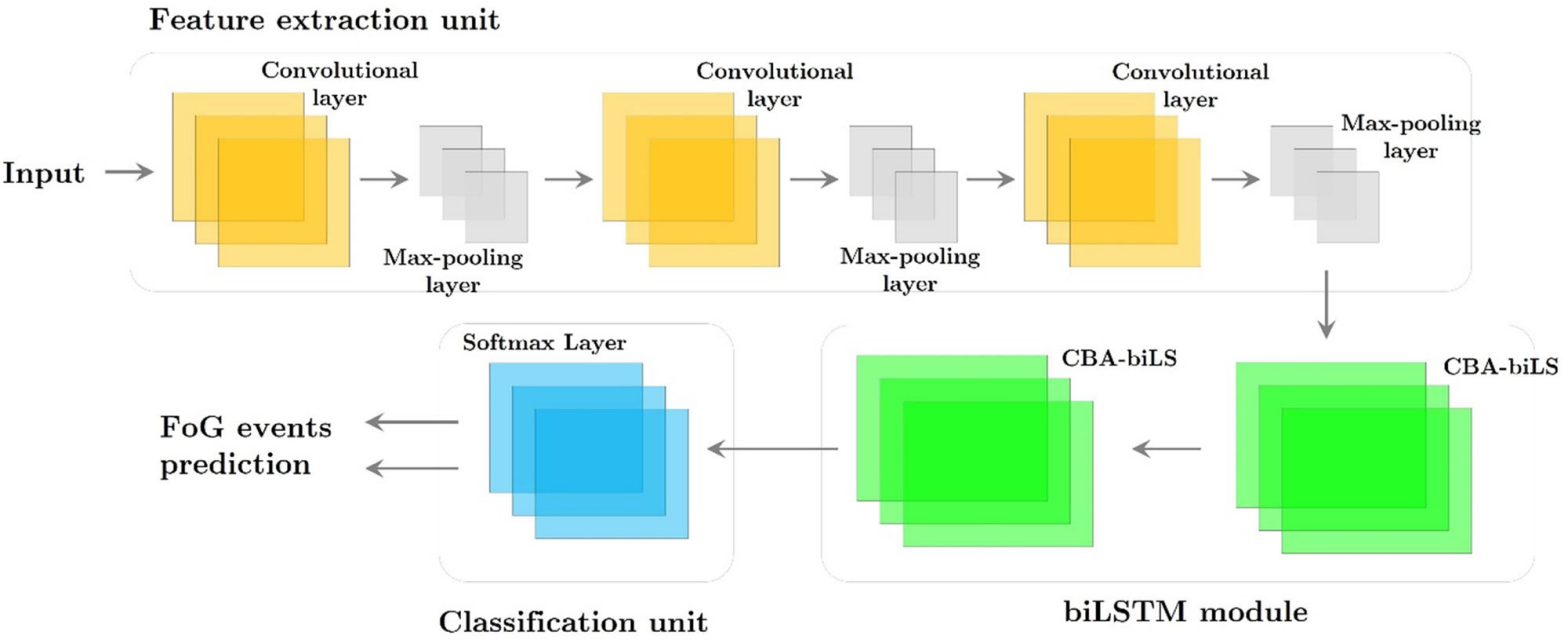
The provided structure offers a comprehensive outline of CBA‐BiLSTM, a model that integrates convolutional, max‐pooling, and softmax layers. CBA‐biLSTM, convolution bottleneck attention–bidirectional long short‐term memory network; FoG, freezing of gait.

The CBA‐BiLSTM framework is developed using CNNs. As shown in Figure 6, the framework consists of two main components: a three‐group interlaced convolution operation with a max‐pooling operation and a bottleneck attention–BiLSTM module. Through the channels and spatial pathways of the bottleneck attention–BiLSTM model, key properties of main sensor signals are extracted. As a result, the subsequent skeleton will be able to select and represent features more effectively. As a result, a softmax layer provides probabilities of accurately identifying both normal and FoG incidents.

## 4 Experimental Results

Analyzing the results based on the implementation parts of the study methodology is discussed in this section. In order to understand gait walking, we need to examine sensor information.

### 4.1 Dataset

We analyzed data received from the UCI database to identify PD in this study (Bächlin et al. 2010; Bachlin et al. 2009). Data collected from the movement of people in the gait can be used to measure automatic methods to detect movement without disturbance or movement disorder using accelerometer sensors worn on the legs and thighs. Label number 2 refers to mild PD, whereas label number 1 refers to severe PD. Accordingly, 237 samples were collected from the lower limbs, joints, thighs, and legs, based on the sensors desired.

**FIGURE 7:**
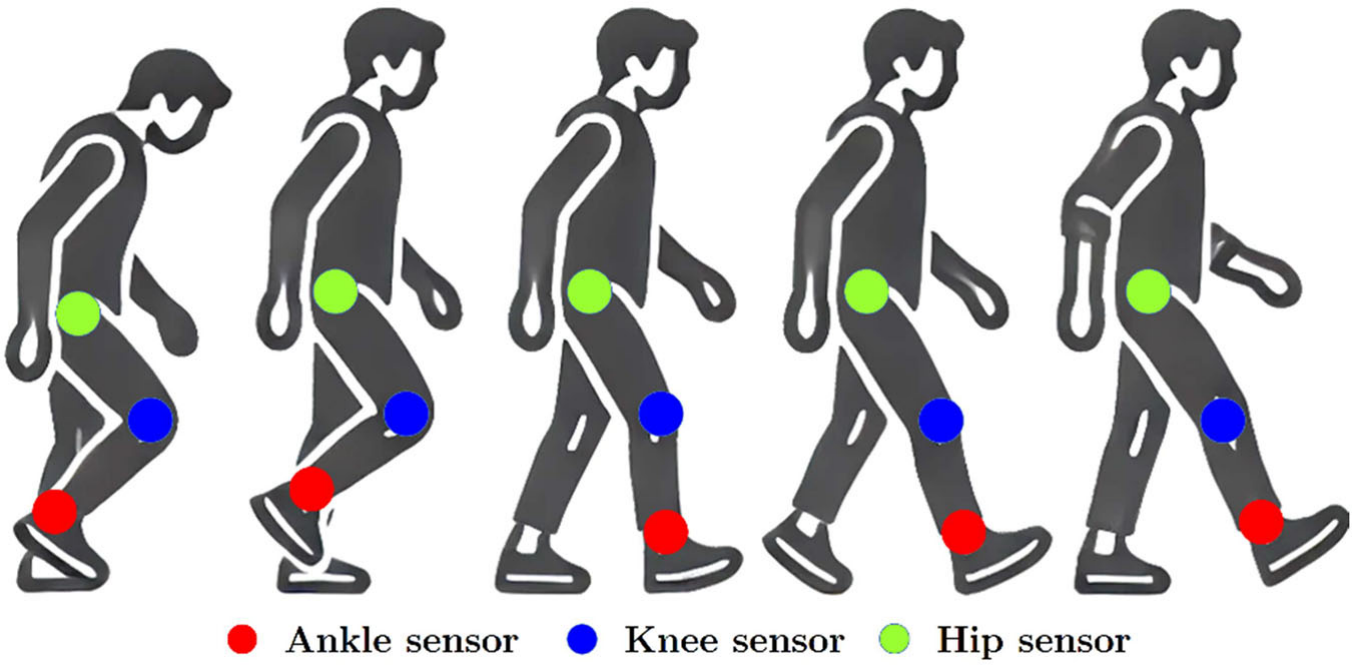
A detection of PD was performed by observing the accelerations of ankle, thigh, and trunk movements during gait movements.

**FIGURE 8:**
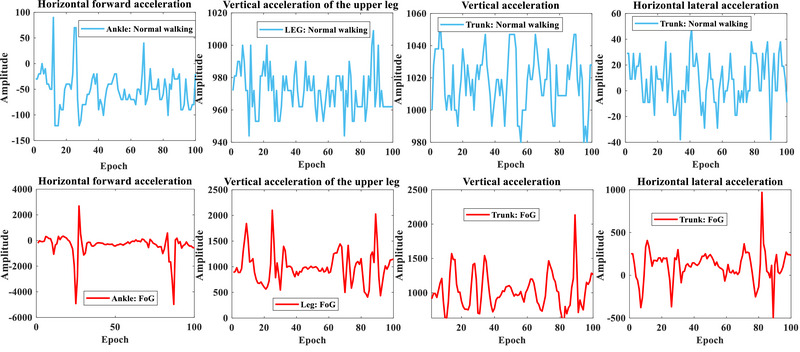
A detection of PD was performed by observing the accelerations of ankle, thigh, and trunk movements during gait movements. FoG, freezing of gait.

The samples were collected from areas related to the lower limbs, joints, thighs, and legs. There have been many movements and signals collected, and because the signals are multi‐channel, in total, 332,355 freezing states and 3 million normal movement states have been categorized. A general characteristic of the motor part has been collected, which is due to the number of channels used to collect data from the ankle joints, thighs, and other motor parts of the lower limbs. In order to better classify and reduce the computational complexity, relevant channels can be included in the channel selection process and designed from their dimensions. According to Figure 7, ankle, thigh, and trunk movements were recorded based on three accelerations in body position. Various acceleration directions were received, including horizontal forward, vertical, and horizontal lateral. For normal walking and FoG, Figure 8 illustrates some samples of channel signals, such as horizontal forward acceleration of the ankle, vertical acceleration of the upper leg, and horizontal lateral acceleration of the trunk.

The ground truth for FoG in the Daphnet dataset was meticulously established through a combination of clinical observations and synchronized video recordings. During data collection, patients were monitored by clinical experts, and their movements were captured in real‐time using wearable sensors placed on the ankle, thigh, and trunk, alongside video documentation. Expert annotators carefully reviewed the video recordings and corresponding sensor data to identify and label FoG episodes, ensuring high precision and alignment with observed events. This process was guided by established clinical protocols for FoG identification and further validated through inter‐rater reliability assessments, where multiple experts independently confirmed the accuracy of annotations. This rigorous methodology ensures the reliability of the dataset as a robust foundation for research into PD and its motor symptoms. The dataset contains approximately 332,355 freezing states and 3 million normal movement states, offering a robust framework for training and evaluation.

### 4.2 Setting

By focusing on a smaller subset of the available initial channels, the optimal option was selected to reduce the processing cost of the initial dataset without affecting the channels themselves. Following the submission of a certain number of channels to the *k*‐NN classification, the accuracy of the classification is evaluated and compared to prior classification iterations. On the basis of the algorithm's repetition, we determined which of 10 alternative network topologies provided the CBA‐BiLSTM with the lowest root mean square error (RMSE) result.

Frequently, the model is tuned by adjusting the number of iterations and learning rates for each layer. During fine‐tuning, between 100 and 500 iterations were performed. Additionally, each of the 10 training groups in the pre‐training session received 100 repetitions of training. In order to determine the efficiency of the network, the RMSE was used. The maximum number of epochs during the advanced training phase is limited to 300. Empirical evidence shows that the initial learning rate is 0.001. When the number of iterating epochs is less than or equal to 100, the learning rate remains constant in order to facilitate convergence. The learning rate decreases by 0.1 for every 10 iterations subsequent to reaching the 100th iteration. In order to achieve a satisfactory equilibrium between coverage and performance, a batch size of 128 has been determined as the optimal choice. Furthermore, units, batch_size, epoch, loss function of the integrated network, and optimizer were set to 64, 128, 200, mean square error (MSE), and Adam optimizer, respectively.

### 4.3 Evaluations

Using the confusion matrix for a binary classifier, we can compute the metrics used for evaluating the classifier. The metrics include accuracy, precision, sensitivity, F‐measure, and specificity. Furthermore, microaveraging (microAVG) weights each category equally, whereas macroaveraging (macroAVG) does not. Three different scenarios are examined in Table 1, illustrating the performance of the proposed model. We examined conditions for applying low, medium, and high layers in Table 1.

**TABLE 1:** Three methods are compared in this table: bottleneck attention–bidirectional long short‐term memory network (Bottleneck attention–BiLSTM), convolutional neural network–BiLSTM (CNN‐BiLSTM), and convolution bottleneck attention–BiLSTM (proposed method).

Model	Metric	Healthy (Class 1)			PD (Class 2)			macroAVG			microAVG		
		Exp. 1	Exp. 2	Exp. 3	Exp. 1	Exp. 2	Exp. 3	Exp. 1	Exp. 2	Exp. 3	Exp. 1	Exp. 2	Exp. 3
Bottleneck attention–BiLSTM	Precision	100	100	100	82.02	81.006	82.73	91.01	90.53	91.36	98.25	98.16	98.32
	Sensitivity	98.10	98.00	98.17	100	100	100	99.05	99.00	99.08	98.25	98.16	98.32
	Specificity	100	100	100	98.10	98.00	98.17	99.05	99.00	99.08	98.25	98.16	98.32
	Accuracy	98.25	98.61	98.32	98.25	98.16	98.32	98.25	98.16	98.32	98.25	98.16	98.32
	F‐score	99.04	98.99	99.08	90.12	89.54	90.55	94.58	94.26	94.81	98.25	98.16	98.32
Convolution ‐BiLSTM	Precision	100	100	100	88.45	88.58	89.15	94.22	94.29	94.57	98.87	98.89	98.94
	Sensitivity	98.77	98.78	98.84	100	100	100	99.38	99.39	99.42	98.87	98.89	98.94
	Specificity	100	100	100	98.77	98.78	98.84	99.38	99.39	99.42	98.87	98.89	98.94
	Accuracy	98.87	98.89	98.94	98.87	98.89	98.94	98.87	98.89	98.94	98.87	98.89	98.94
	F‐score	99.38	99.39	99.42	93.87	93.94	94.26	96.62	96.66	96.84	98.87	98.89	98.94
Convolution bottleneck attention–BiLSTM	Precision	100	100	100	96.50	95.03	96.43	98.25	97.51	99.80	99.66	99.51	99.65
	Sensitivity	99.62	99.46	99.60	100	100	100	99.81	99.73	99.80	99.66	99.51	99.65
	Specificity	100	100	100	99.62	99.46	99.61	99.81	99.73	99.80	99.66	99.51	99.65
	Accuracy	99.66	99.51	99.64	99.66	99.51	99.65	99.66	99.51	99.65	99.66	99.51	99.65
	F‐score	99.81	99.73	99.80	98.21	97.45	98.18	99.01	98.59	98.99	99.66	99.51	99.65

Abbreviation: PD, Parkinson's disease.

When more layers are used, classification may be improved slightly depending on the PD signals. Three separate experiments were conducted for each test to ensure CV. The three structures have been fine‐tuned, and the number of parameters has been prevented from increasing. In all three structures, the evaluation criteria are similar, and the results of the experiments are not significantly different. However, accuracy tends to improve when the channel and spatial attention fusion is employed. In comparison with other CNN‐ or BiLSTM‐based models with many parameters or computational complexities, the CBA‐BiLSTM method results in optimal outputs using the appropriate number of layers and parameters. As a result of the LSTM structure, the accuracy of predicting the classes (healthy and Parkinson's) has improved. However, it's the combination of convolution and bottleneck attention that makes things better.

Table 2 shows the comparison of all experimental results between our proposed method and the common DL methods, for example, LSTM, BiLSTM, and CNN‐BiLSTM, on the multi‐modal PD dataset from gait signals. It is clear that our method has achieved more efficient experimental results in all cases. For the classification task of healthy and PD, our method achieves the highest accuracy rate of 99.66%, 99.45%, and 99.17%, respectively, at the high, moderate, and low number of applied selected channels. Table 2 provides estimates of accuracy, sensitivity, specificity, F‐score, and Kappa. On the basis of the movement of healthy and PD people, the checking conditions are the same as in the previous calculations. In response analysis, convolution models and BiLSTM methods have better responses than other architectures. Accordingly, nine main channels are included; the lowest number is 3 channels, the highest number is 8 to 9 channels, and the moderate number is 5 to 6 channels.

**TABLE 2:** On the multi‐modal Parkinson's disease (PD) dataset from gait signals, this table compares all experimental results between our proposed method (convolution bottleneck attention–bidirectional long short‐term memory network [CBA‐BiLSTM]) and the common deep learning methods.

No. channels	Method	Accuracy (%)	Sensitivity (%)	Specificity (%)	F‐score (%)	Kappa (%)	Computational complexity
High	LSTM	98.46	99.58	85.55	99.15	90.69	High
	BiLSTM	98.55	99.89	86.02	99.20	91.20	High
	CNN‐BiLSTM	99.02	99.96	90.30	99.46	94.15	High
	Proposed	99.66	99.62	100	99.81	98.03	Moderate
Moderate	LSTM	98.76	99.87	88.38	99.32	92.57	Moderate
	BiLSTM	98.85	99.94	88.76	99.37	93.12	Moderate
	CNN‐BiLSTM	99.05	99.90	91.17	99.48	94.38	High
	Proposed	99.45	99.39	100	99.69	96.76	Moderate
Low	LSTM	98.00	99.78	81.43	98.90	87.69	Moderate
	BiLSTM	98.51	99.89	85.64	99.18	90.97	Low
	CNN‐BiLSTM	98.68	99.86	87.64	99.27	92.05	Moderate
	**Proposed**	99.17	99.09	100	99.54	95.10	**Low**

*Note*: Best performance is indicated by boldface.

Abbreviation: CNN, convolutional neural network.

The effectiveness of the channels selected by the CBA‐BiLSTM is shown in Table 3 based on their accuracy in helping classification. Meanwhile, Channels 6 and 7 have been most effective in diagnosing PD.

**TABLE 3:** According to the accuracy obtained from each test, this table shows the most effective channels.

Selected channels	Percentage of channels	No. channels	Accuracy (%)
5	∼12	Low	98.54
5,8	∼23	Low	99.13
5,8,9	∼34	Low	99.32
5,7,8,9	∼45	Moderate	99.46
5,7,8,9,1	∼56	Moderate	99.71
5,7,8,9,1,6	∼67	Moderate	99.82
5,7,8,9,1,6,4	∼78	Moderate	99.75
5,7,8,9,1,6,4,3	∼89	High	99.52
5,7,8,9,1,6,4,3,2	∼100	High	99.39

It is demonstrated that the two sensors that measure vertical acceleration of the upper leg and vertical acceleration of the trunk are highly effective for identifying PD and FoG. Among the channels that are called in this table are (1) horizontal forward acceleration of the ankle, (2) vertical acceleration of the ankle, (3) horizontal lateral acceleration of the ankle, (4) horizontal forward acceleration of the upper leg, (5) vertical acceleration of the upper leg, (6) horizontal lateral acceleration of the upper leg, (7) horizontal forward acceleration of the trunk, (8) vertical acceleration of the trunk, and (9) horizontal lateral acceleration of the trunk.

**FIGURE 9:**
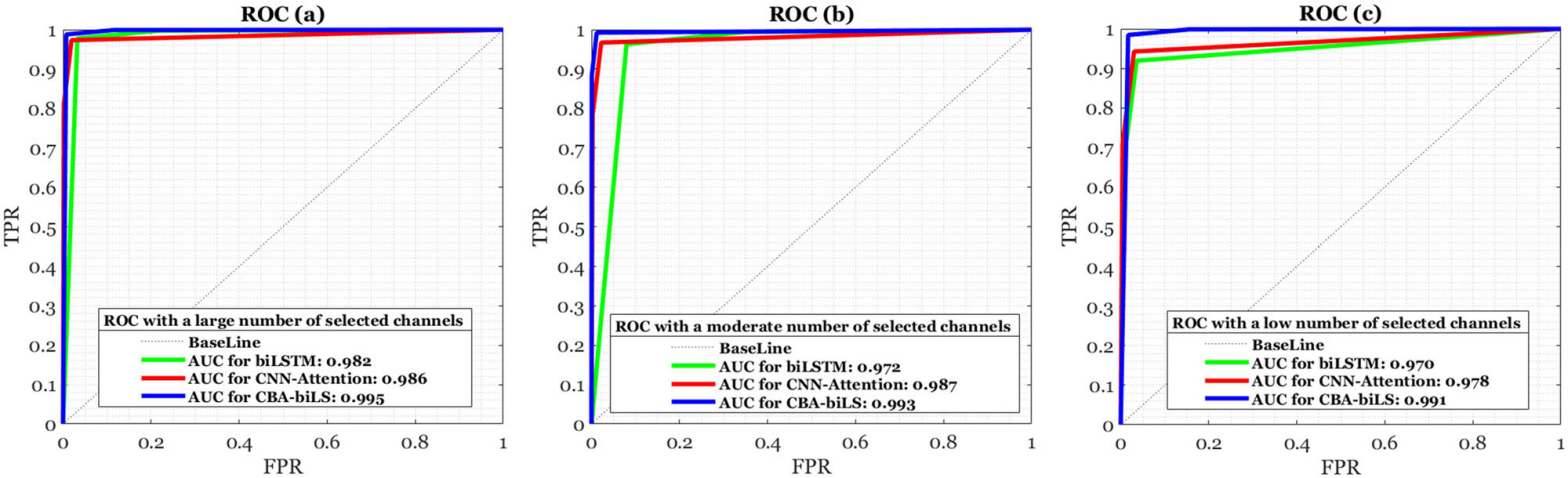
This figure compares the performance of two strong approaches with the proposed method based on ROC curves. AUC, area under the curve; biLSTM, bidirectional long short‐term memory network; CBA, convolution bottleneck attention; CNN, convolutional neural network; ROC, receiver operating characteristic.

The area under the ROC curve estimates the test's overall utility, with larger areas indicating greater value, which can help determine which tests are most beneficial. For various PD detection structures, Figure 9 illustrates the ROC curve AUC. The analyzed signals are a set of unseen signals, and the low AUC decrease of the proposed method shows its robustness: (1) AUC comparison of three methods considering a large number of channels, (2) AUC comparison of three methods considering a moderate number of channels, and (3) AUC comparison of three methods considering a low number of channels. ROC curves have been used to extract FoG from motion, and their performance has been evaluated over a wide range of signals. In addition to evaluating the states of people's movement at the gait, other signal circumstances are also considered.

Furthermore, Figure 9 shows the robustness of the proposed method when it comes to reducing the number of channels. Therefore, the AUC of FoG detection does not drop much when the number of channels is reduced (e.g., about a 0.4% drop in identification accuracy), whereas the AUC of two other methods drops from 1% to 1.5%. As a result of the improved design of the proposed framework, the AUCs were uniformly high. Figure 9 depicts the ROC curve for various random signals of human movement, including those of healthy and PD patients, and their comparison to the proposed method.

## 5 Discussion

A fundamental justification for using the proposed method to identify FoG events in received signals from people's movement in gait is its capability to categorize signals adequately into two or more classes as intensity detection of PD. The physician will be able to make an accurate diagnosis while also being able to monitor the patient continuously throughout the day. By using a better decision‐making model, signals are monitored, analyzed, and classified. In the following sections, we discuss each approach in more detail, along with its justifications and outcomes.

### 5.1 Error in Monitoring FoG

The proposed method in this article is accurate when it comes to PD diagnosis, but it encounters a high rate of false positive errors, which translates to a lower level of specificity than sensitivity. A physician makes the final decision not to perform a PD during signal monitoring; however, if a false positive is detected.

**FIGURE 10:**
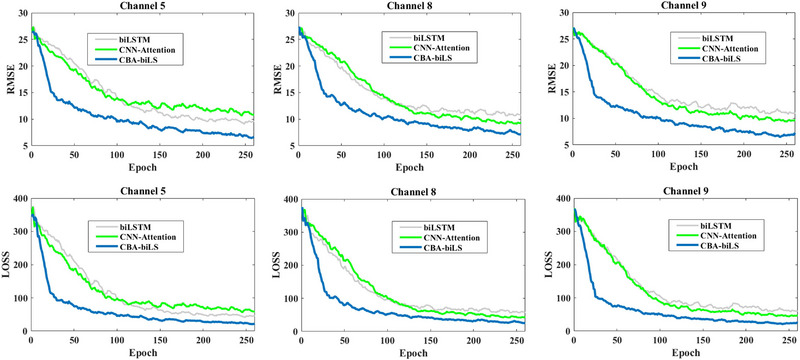
On the basis of RMSE and LOSS, this figure compares the almost real‐time error of monitoring FoG for three effective channels with two DL structures. biLSTM, bidirectional long short‐term memory network; CBA, convolution bottleneck attention; CNN, convolutional neural network; RMSE, root mean square error.

Due to the large number of signals in which no PD events occurred, our model is trained based on the aggregation of all signals; therefore, false positive error is inevitable. When the symptoms of PD exceed a physician's threshold for diagnosis, the diagnosis is confirmed as PD. In the worst cases, some tests have specificity greater than 95%, but signal monitoring is more helpful to a specialist physician than other methods. Figure 10 shows an almost real‐time error in monitoring FoG for three effective channels compared to two DL structures based on RMSE and LOSS criteria. The CBA‐BiLSTM and similar models are trained using data collected from sensors worn by Parkinson's patients for Parkinson's monitoring and FoG prediction. In order to evaluate the model's accuracy in predicting FoG episodes, metrics such as error, LOSS, and RMSE were used. The results indicate that Parkinson's monitoring was appropriate for random sets of signals with and without PD events. Therefore, we can conclude that the proposed method has been used for what purpose and is efficient for detecting FoG through three effective channels.

### 5.2 Channel Selection

**FIGURE 11:**
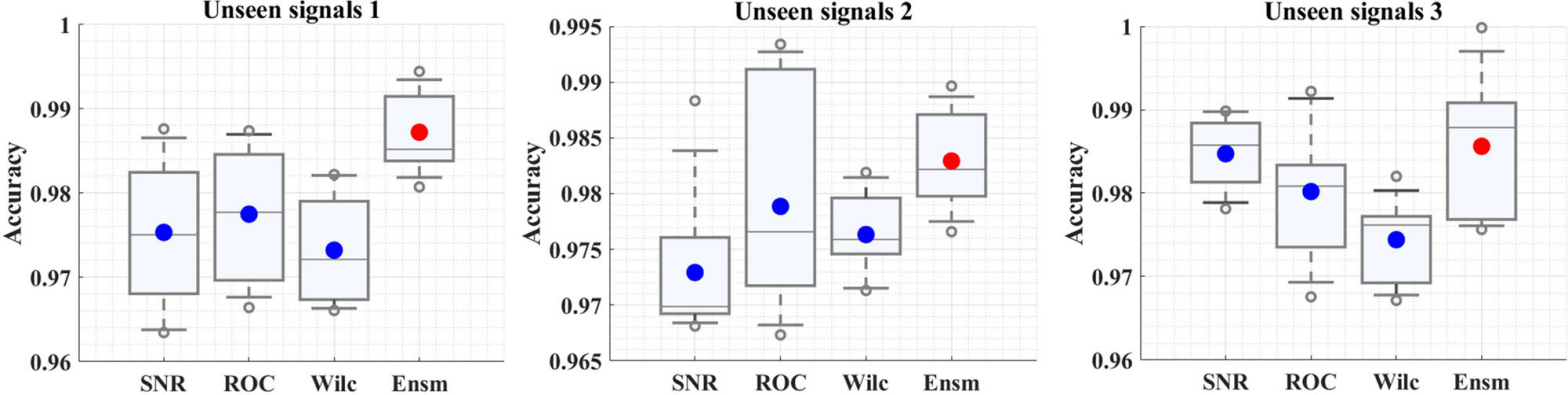
When using three unseen signals in channel selection, the ensemble (Ensm) model performs better than other similar approaches. ROC, receiver operating characteristic; SNR, signal‐to‐noise ratio.

According to our study, ensemble channel selection can significantly increase CBA‐BiLSTM model accuracy in FoG detection while reducing data dimensionality, resulting in more reliable and efficient decision‐making systems that can be applied to real‐world scenarios with greater confidence. Experimentally, ensemble channel selection significantly improved model accuracy compared to traditional channel selection methods, such as the each and every method or other channel‐based methods. Our ensemble channel selection method was particularly effective when dealing with high‐dimensional data, where traditional channel or feature selection methods struggle due to computational complexity. As illustrated in Figure 11, the ensemble model (Ensm) provides better channel selection than other similar approaches when using three sets of unseen signals. There are narrow minimum and maximum distances for ideal channel selection for the three FoG signal models. Furthermore, the suggested feature selection approach is less accurate than the confidence interval for selecting efficient channels. FoG detection using the suggested approach consistently outperforms expert assessment (*p* value < 0.01), and it has small standard deviations.

Notably, the sensor configuration used in this study, consisting of placements on the ankle, leg, and trunk, was chosen based on its established efficacy in capturing critical features of gait and FoG events in PD. Lower limb placements, particularly on the ankle and shank, are widely regarded as optimal for detecting FoG, as they directly measure movement patterns most affected during episodes of freezing. Studies have consistently demonstrated that sensors in these locations provide reliable data for FoG detection with minimal redundancy (Azevedo et al. 2014; Moore et al. 2013).

Additionally, this configuration minimizes patient discomfort and system complexity, which are critical for wearable applications. Increasing the diversity of sensor placements, such as including wrist‐ or head‐mounted sensors, may offer complementary information but would increase the cost, computational demands, and user burden, potentially limiting practical adoption (Mazilu, Blanke, and Tröster 2015). Despite the limited diversity, our system achieved high sensitivity and specificity, demonstrating its robustness and ability to capture the critical movement characteristics necessary for accurate FoG detection.

Furthermore, many established protocols in the field emphasize the importance of simplicity and patient comfort in sensor placement, as systems requiring fewer sensors or those targeting the lower limbs are often preferred in clinical and real‐world settings (Silva de Lima et al. 2017). This approach ensures practicality while maintaining high diagnostic performance. Future work will explore the integration of additional sensor placements, such as the wrist or lumbar region, to further investigate their potential to enhance detection accuracy without compromising usability and efficiency.

### 5.3 Computational Complexity and False Positive Optimization

The comparison of computational performance across methods, as shown in Table 4, highlights the training and testing time disparities driven by architectural differences. Among the evaluated models, CNN‐GRU is the fastest in training (∼2 h 15 m) due to its simplified recurrent unit design, whereas transformer‐based models take significantly longer (∼12 h 30 m), attributed to their self‐attention mechanisms and large parameter sets. The proposed CBA‐BiLSTM balances these extremes, with a training time of ∼5 h 45 m, showcasing efficiency without sacrificing feature extraction quality. Additionally, during testing, CBA‐BiLSTM demonstrates moderate inference time, outperforming attention‐heavy models like Attention‐CNN‐LSTM and transformer‐based models, which exhibit high testing overheads. These results make the proposed method particularly advantageous for real‐time applications.

**TABLE 4:** Comparison of training time, testing time, parameter count, and suitability for wearable applications, with emphasis on the computational efficiency and reduced complexity of the proposed convolution bottleneck attention–bidirectional long short‐term memory network (CBA‐BiLSTM) model.

Method	Training time	Testing time	Number of parameters (million)	Suitability for wearables	Performance
CNN‐LSTM	4 h 30 m 0 s	Moderate (LSTM slows down inference slightly)	∼5	Moderate (may require optimization for wearables)	High (strong for spatial and temporal extraction)
CNN‐GRU	2 h 15 m 0 s	Lower (GRU simplifies inference)	∼3	High (lightweight and efficient)	Moderate‐to‐high (trade‐off in accuracy)
Attention‐CNN‐LSTM	6 h 0 m 0 s	High (attention introduces overhead in testing)	∼6	Moderate (attention increases computation req.)	High (attention enhances feature selection)
Transformer‐based models	12 h 30 m 0 s	Very high (self‐attention increases latency)	20+	Low (high resource demands)	Very High (excels in long‐range dependencies)
CBA‐BiLSTM (proposed)	5 h 45 m 0 s	Moderate (efficient inference with optimized arch.)	∼2–3	High (optimized for lower computation)	High (optimized feature extraction and BiLSTM)

Abbreviations: CNN, convolutional neural network.

Table 4 further illustrates the parameter efficiency and suitability of each method for wearable applications. Transformer‐Based Models, with over 20 million parameters, demand high computational resources and are unsuitable for devices with limited processing power. In contrast, the proposed CBA‐BiLSTM maintains a parameter count of ∼2–3 million, significantly lower than CNN‐LSTM (∼5 million) and Attention‐CNN‐LSTM (∼6 million). This parameter efficiency, coupled with reasonable training and testing times, makes CBA‐BiLSTM an optimal choice for power‐constrained environments, such as wearable devices used in clinical and home settings.

Lastly, Table 4 emphasizes the trade‐offs between performance and computational complexity. Although all models achieve high accuracy, the proposed CBA‐BiLSTM leads with an accuracy of 99.88%, striking an ideal balance between performance and computational efficiency. Transformer‐based models, though accurate (99.34%), are impractical for real‐world scenarios due to their high complexity and resource demands. Similarly, although CNN‐GRU and CNN‐LSTM are computationally simpler, they show slightly reduced accuracies (95.32% and 97.48%, respectively). The inclusion of bottleneck attention in CBA‐BiLSTM enhances its ability to prioritize critical features while minimizing redundancy, positioning it as the most efficient and effective solution for real‐time FoG detection.

The proposed model, optimized for low latency and computational efficiency (∼2–3 M parameters), is well suited for real‐time applications. Future work will focus on deploying the model on wearable devices and validating its performance in clinical and home settings, assessing latency, computational demands, and user experience. Additionally, a real‐time feedback loop for patients and caregivers will be developed to enhance its practical utility in managing FoG.

In addition, the analysis of Table 5 highlights the distinct advantage of the proposed CBA‐BiLSTM in minimizing the false positive rate (FPR) compared to other models. With an estimated FPR ranging from low to moderate, this method demonstrates robust specificity, attributed to the integration of the bottleneck attention mechanism. This mechanism filters irrelevant features while enhancing critical ones, significantly reducing noise and misclassifications. Unlike models such as CNN‐LSTM, which rely on temporal dependencies and may misinterpret noise as meaningful patterns, CBA‐BiLSTM effectively mitigates such risks. Similarly, CNN‐GRU, with its simpler temporal modeling, achieves moderately low FPR but lacks the refinement provided by attention mechanisms, limiting its performance in more complex scenarios. On the other hand, transformer‐based models, while excelling in long‐range dependencies, exhibit a higher FPR due to their sensitivity to input noise and inherent complexity, which makes them less suited for real‐world applications requiring high specificity.

**TABLE 5:** Estimated false positive rate across various methods, emphasizing the comparative advantage of the proposed convolution bottleneck attention–bidirectional long short‐term memory network (CBA‐BiLSTM) in achieving lower false positives through its bottleneck attention mechanism.

Method	False positive rate (estimated)	Reasoning
CNN‐LSTM	Moderate	LSTM's reliance on temporal patterns can lead to higher sensitivity but more false positives
CNN‐GRU	Moderate‐to‐low	Simplified temporal modeling in GRU reduces overfitting and false positives
Attention‐CNN‐LSTM	Moderate	Attention improves feature focus but may amplify misclassifications in noisy data
Transformer‐based models	Moderate‐to‐high	High complexity and sensitivity to input noise can lead to more misclassifications
CBA‐BiLSTM (proposed)	Low‐to‐moderate	Bottleneck attention reduces irrelevant features, improving specificity

Abbreviation: CNN, convolutional neural network.

The superior performance of CBA‐BiLSTM extends beyond reduced FPR to its practicality for real‐time and wearable applications. With moderate computational complexity and a compact parameter set (∼2–3 M), this method balances sensitivity and specificity more effectively than other architectures. Models like Attention‐CNN‐LSTM, while leveraging attention mechanisms, still exhibit moderate FPR due to their reliance on noisy data and additional overhead. The proposed model's ability to maintain high sensitivity without compromising specificity makes it ideal for real‐world scenarios, such as clinical monitoring or home‐based applications, where false positives can lead to unnecessary alerts and reduced user trust. This balance between computational efficiency and accuracy, as evidenced in Table 5, positions CBA‐BiLSTM as the optimal solution for FoG detection in PD, addressing both technical and practical challenges effectively.

### 5.4 Comparison

FoG can be identified using general‐purpose or patient‐specific datasets from PD using several different approaches. Validation, also known as separation of training and test data, is the fundamental criterion for evaluating ML algorithm models (Camps et al. 2018; Bachlin et al. 2009; Assam and Seidl 2014; Rezvanian and Lockhart 2016; Ahlrichs et al. 2016; Pham et al. 2017; Polat 2019; Hausdorff, Balash, and Giladi 2003; Delval et al. 2010; Uchitomi et al. 2023) (see Table 6). The development of person‐specific models for PD patients, however, requires large quantities of data. Several small datasets are also tested by CV (Hu et al. 2019; Bachlin et al. 2009; Hausdorff, Balash, and Giladi 2003; Borzì et al. 2023; Mikos et al. 2017; Zhang and Gu 2019; Borzì et al. 2021; Ashfaque Mostafa et al. 2021; Pardoel et al. 2021). In this process, algorithms derived from ML are used, from basic threshold approaches to complicated algorithms like SVM and CNN. There are several straightforward classifiers that can be constructed rapidly but yield subpar results, including the learning model (Aşuroğlu et al. 2018) and the freezing index (FI) threshold (Moore, MacDougall, and Ondo 2008). In a number of research studies, performance measures have been extracted from Fourier transforms (Bachlin et al. 2009; Pham et al. 2017; Delval et al. 2010; Aşuroğlu et al. 2018).

**TABLE 6:** Our CBA‐BiLSTM (convolution bottleneck attention–bidirectional long short‐term memory network) model is compared with quantitative metrics of reported methods using several datasets.

Author	Method	Accuracy	Precision	Sensitivity	Specificity
Hu et al. (2019)	Graph CNN	82.34%	—	81.90%	82.44%
Bikias et al. (2021)	CNN	—	83.00%	—	88.00%
Yuan and Chakraborty (2020)	RNN‐LSTM	94.70%	—	—	—
Murad and Pyun (2017)	Cascade‐LSTM	94.10%	87.70%	78.90%	—
Xia et al. (2018)	CNN	80.70%	69.29%	—	90.60%
San‐Segundo et al. (2019)	MLP‐CNN	—	—	93.10%	75.00%
Camps et al. (2017)	4 layers‐CNN	—	—	79.00%	80.00%
Camps et al. (2018)	8 layers‐CNN	89.10%	—	91.90%	89.50%
Kim et al. (2018)	CNN	—	—	93.80%	90.10%
Zhang and Gu (2019)	LSTM	90.60%	—	91.80%	91.30%
Ashfaque Mostafa et al. (2021)	CNN‐LSTM	98.50%	—	98.54%	97.91%
Masiala (2017)	LSTM‐RNN	—	—	79.00%	80.00%
El‐ziaat, El‐Bendary, and Moawad (2022)	CNN	96.80%	96.30%	95.40%	96.10%
Mekruksavanich, Jantawong, and Jitpattanakul (2022)	SE‐DeepConv	95.65%	95.57%	95.65%	—
Our model	**Channel selection and CBA‐BiLSTM**	**99.88%**	—	**99.99%**	**98.89%**

Abbreviations: CNN, convolutional neural network; MLP, multilayer perceptron; RNN, recurrent neural network.

Moreover, data collection windows might last anywhere from 0.2 to 20 s. Most of these types of models are exclusive to one individual, so they require customization. ML models based on random forest trees and AdaBoost trees are most effective for 1 s time windows. It has been discovered that DL approaches provide comprehensive solutions to problems. In contrast, ML and traditional methods like handmade models require parsing issue statements into their component components and reassembling them. Due to FoG episodes being unexpected and symptoms being diverse, person‐specific models require a lot of data to train. Constrained semi‐supervised learning can instead be used to create specialized FoG detection algorithms. The most effective way to define FoG episodes is by visually marking and labeling them. CNN, stacked auto‐encoders, and LSTM models are most effective at detecting FoG events in patients with highly variable movement patterns (Ashfaque Mostafa et al. 2021). For training and validating this method on a variety of patient populations, a large dataset is required. Due to the small sample size of current research, these ML models may not be sufficient. Table 6 demonstrates that the proposed model outperforms other state‐of‐the‐art methods for classifying FoG events. As previously discussed, the proposed model features an innovative architecture that integrates a bottleneck attention module into a traditional BiLSTM framework. This enhancement enables the model to concentrate on the most critical features within the movement signal data, resulting in unprecedented accuracy in predicting FoG. Furthermore, this approach represents the first documented application of a CBA‐BiLSTM specifically designed for PD classification and FoG detection.

When compared to other reported methods, the proposed model achieves a remarkable accuracy of 99.88%, significantly higher than the best previously reported accuracy of 98.50% by Mostafa et al. using a CNN‐LSTM. Furthermore, this model demonstrates an exceptional sensitivity of 99.99%, surpassing all other methods, including the 98.54% sensitivity achieved by Ashfaque Mostafa et al. (2021). Additionally, the model's specificity is 98.89%, which is also notably higher than the specificity achieved by other methods.

These metrics underscore the superiority of this method in terms of both accuracy and sensitivity, providing a more reliable and efficient solution for detecting FoG in PD patients. Unlike previous approaches, this model not only improves prediction accuracy but also enhances the practical applicability of FoG detection by focusing on the most relevant features through its innovative architecture. This method is unique and unprecedented, achieving a level of accuracy in sensor data analysis during movement that has not been seen before in Parkinson's detection research.

### 5.5 Limitations and Future Directions

There are, however, some flaws in this research. A primary problem is the inadequacy of sample sizes. Moreover, appropriate channel selection could not be optimized alongside other approaches, which was one of its disadvantages. Ultimately, there were only three different sensor nodes used in this study for gait analysis, but the detection rate of FoG events was low as a result of the limited number of channels. DL structures have been created that will enable future research to employ low sensor nodes for capturing patient motions and analyzing kinematics more deeply.

This study utilized a publicly available dataset, recognized for its high‐quality annotations but limited in demographic and clinical diversity. Despite this, extensive experiments on diverse subsets of the dataset revealed consistent performance, with minimal variance in accuracy, sensitivity, and specificity, highlighting the model's robustness and generalizability. To further enhance diversity, future work will focus on expanding the dataset through collaborations with clinical centers, incorporating participants across varied demographics and PD stages. Additionally, multicentric data integration will ensure broader applicability and reliability in real‐world scenarios.

Although the dataset used in this study is relatively small, it is a unique and highly specialized dataset specifically designed for FoG detection in PD. The dataset contains a substantial number of individual samples and provides high‐quality annotations, enabling robust model development. Additionally, although the processed data was used for this work, the raw signal data could be windowed to create augmented datasets, further expanding the dataset's utility. In its current form, the dataset demonstrates excellent separability for FoG detection, as confirmed by the consistent performance observed across multiple DL models and the minimal variance in accuracy over repeated experiments. These observations suggest strong generalizability of the proposed method. Nevertheless, future efforts will focus on validating the model using larger and more diverse datasets, including augmented versions, to further enhance its robustness and real‐world applicability.

Moreover, although the proposed method demonstrates remarkable sensitivity (99.99%) and high specificity (98.89%), a slightly elevated FPR was observed. This behavior is a common trade‐off in high‐sensitivity detection systems, especially in critical healthcare applications where the priority is ensuring no critical events, such as FoG, are missed. In this context, false positives, although not ideal, are less detrimental than false negatives, as missing an actual FoG event could lead to significant patient harm.

The high specificity observed (98.89%) suggests that the FPR is not excessive and remains within acceptable limits for practical applications. Additionally, in real‐world use, false positives may prompt additional monitoring or minor interventions, which are preferable to the risks associated with undetected FoG events. Furthermore, our analysis across multiple DL models showed consistent performance with low variance, highlighting the robustness of the method.

Future efforts will focus on refining the model to further reduce false positives without compromising sensitivity, potentially through advanced post‐processing techniques or hybrid approaches that incorporate context‐aware filtering. Moreover, evaluations on larger and more diverse datasets will provide additional insights into optimizing this balance for broader clinical and real‐world scenarios.

## 6 Conclusion

A model for FoG monitoring and classification was developed using the motion signals of PD patients during movement based on the convolution bottleneck attention–BiLSTM or CBA‐BiLSTM architecture. By monitoring and categorizing FoG occurrences using ankle, leg, and trunk sensors, monitoring and categorization of FoG were anticipated. Because each of the sensors’ three locations receives samples from three motion directions, a combination set approach based on channel selection is advantageous. Hence, the vertical accelerations of the upper leg and trunk were the most accurate in identifying FoG. When a large number of points show anomalous movement in persons, we conclude that FoG has occurred. As a consequence, Parkinson's patients are easily identifiable. By improving the DL architecture and using channel selection, false positives were reduced and computing complexity was lowered. Additionally, we did not employ the windowing method as we analyzed the movement samples of individuals individually, which enables movement tracking as well as accurate detection of PD and FoG. Compared to standard DL approaches, the CBA‐BiLSTM significantly improved classification results. Our future plans include applying a real‐time FoG prediction system to confirm the results and examine the effects of feedback stimulation (Supporting Information).

## Author Contributions

**Sara Abbasi**: Conceptualization, investigation, writing–original draft, methodology, data curation. **Khosro Rezaee**: Conceptualization, investigation, writing–original draft, methodology, validation, visualization, writing–review and editing, data curation, supervision, project administration.

## Ethics Statement

The research has ethical approval. The datasets from UCI dataset have been made public to all researchers.

## Consent

The research has research consent from all authors.

## Conflicts of Interest

The authors declare no conflicts of interest.

### Peer Review

The peer review history for this article is available at https://publons.com/publon/10.1002/brb3.70206.

## Supporting information



Supporting Information

## Data Availability

The data are accessible by request from the corresponding author. All the data and materials in this article are real and available. Data are available from the corresponding authors. The following link also provides signals: https://archive.ics.uci.edu/ml/datasets/Daphnet+Freezing+of+Gait.
