# Supplementary material for: Deep Learning–Based Prediction of Freezing of Gait in Parkinson's Disease With the Ensemble Channel Selection Approach
Source: Brain Behav. 2024 Dec 31;15(1):e70206. doi: 10.1002/brb3.70206 (PMC11688057; doi:10.1002/brb3.70206)
Supplement: Supplementary file 1 — Supporting Information [file BRB3-15-e70206-s001.docx]

**Supplementary Materials**


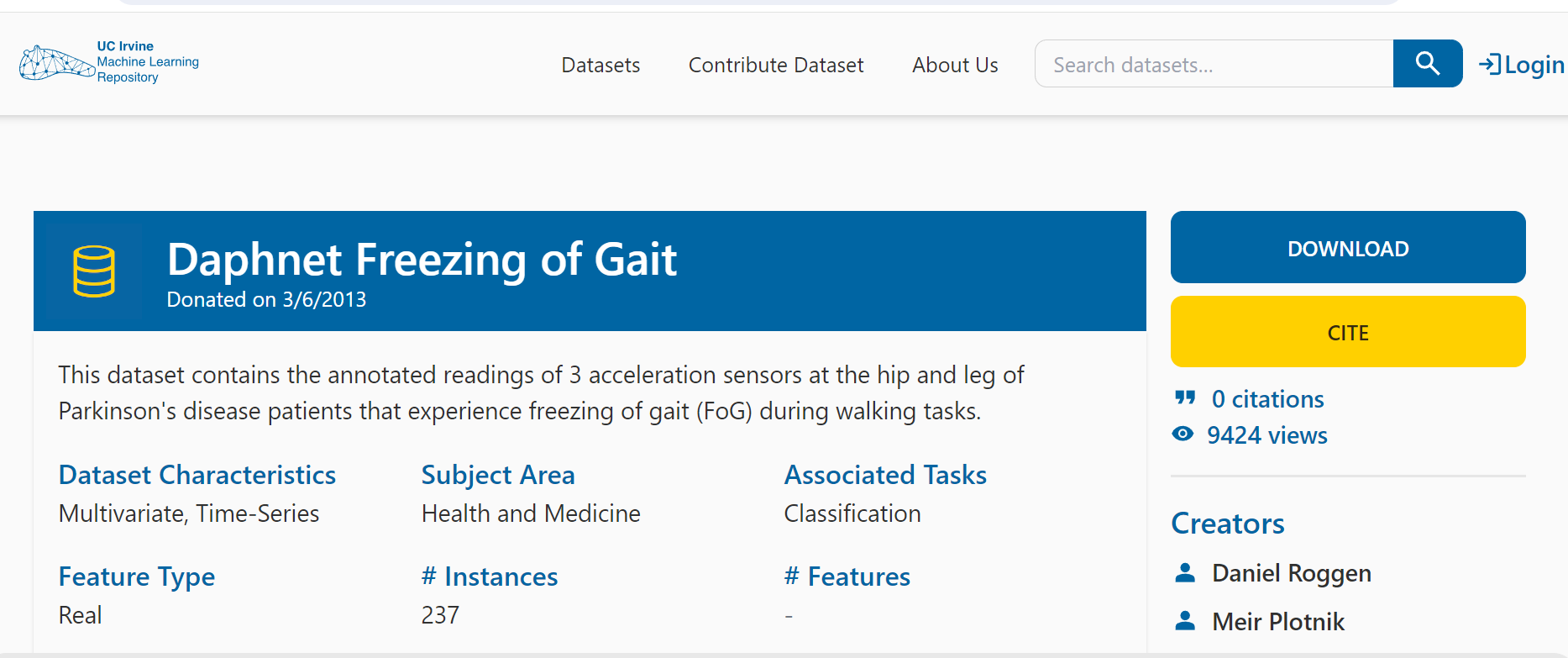


**Data Accessibility**

The data supporting the findings of this study are available upon request from the corresponding author. All data and materials presented in this article are authentic and accessible. Additionally, the Daphnet Freezing of Gait dataset can be accessed via the following link: [UCI Machine Learning Repository - Daphnet Freezing of Gait Dataset](https://archive.ics.uci.edu/ml/datasets/Daphnet+Freezing+of+Gait)

**Additional Information**

The Daphnet Freezing of Gait Dataset is designed to benchmark automatic methods for recognizing gait freeze from wearable acceleration sensors placed on the legs and hip. The dataset was recorded in a laboratory setting with a focus on generating numerous freeze events. Participants performed three types of tasks: straight-line walking, walking with numerous turns, and a more realistic activity of daily living (ADL) task, which involved activities like fetching coffee and opening doors. This dataset is a collaborative effort between the Laboratory for Gait and Neurodynamics at Tel Aviv Sourasky Medical Center, Israel, and the Wearable Computing Laboratory at ETH Zurich, Switzerland. The recordings were conducted at Tel Aviv Sourasky Medical Center in 2008. The study was approved by the local Human Subjects Review Committee and conducted in accordance with the ethical standards of the Declaration of Helsinki.

**Additional Variable Information**

Each file in the dataset comprises data in a matrix format, with each line representing a sample and each column representing a channel. The channels are as follows:

- Time of sample in milliseconds
- Ankle (shank) acceleration - horizontal forward acceleration [mg]
- Ankle (shank) acceleration - vertical [mg]
- Ankle (shank) acceleration - horizontal lateral [mg]
- Upper leg (thigh) acceleration - horizontal forward acceleration [mg]
- Upper leg (thigh) acceleration - vertical [mg]
- Upper leg (thigh) acceleration - horizontal lateral [mg]
- Trunk acceleration - horizontal forward acceleration [mg]
- Trunk acceleration - vertical [mg]
- Trunk acceleration - horizontal lateral [mg]
- Annotation [0, 1, or 2]

The annotations have the following meanings:

- 0: Not part of the experiment (e.g., sensors are being installed on the user or the user is performing activities unrelated to the experimental protocol, such as debriefing).
- 1: Experiment, no freeze (can be any of standing, walking, or turning).
- 2: Freeze.

**Image Information**

- Format: Portable Network Graphic (PNG)
- Bits Per Pixel: 32
- Color: Truecolor with alpha
- Dimensions: 1867 x 783
- Interlaced: Yes
- XResolution: 96
- YResolution: 96
